# Prognostic Utility of Prothrombin Time-International Normalized Ratio, Interleukin-6, and High-Density Lipoprotein Levels in Patients With Severe Sepsis

**DOI:** 10.7759/cureus.92360

**Published:** 2025-09-15

**Authors:** Manoj Kumar Kurmana, Maniram Kumhar, Ravindra Kumar Tiwari, Harsh Tak, Ravi Gaur

**Affiliations:** 1 Internal Medicine, Jawaharlal Nehru Medical College, Ajmer, IND

**Keywords:** biomarkers, high-density lipoprotein (hdl), intensive care, interleukin-6 (il-6), mortality, prognosis, prothrombin time-international normalized ratio (pt-inr), risk stratification, sepsis

## Abstract

Background

A foremost cause of mortality in intensive care units is sepsis, especially in resource-limited nations such as India, where delays in patient presentations and limited diagnostic facilities pose common challenges. While conventional severity scores such as Sequential Organ Failure Assessment (SOFA) and Acute Physiology and Chronic Health Evaluation II (APACHE II) are helpful, the multiple assessments required and labor-intensive calculations create an impractical situation for some clinicians. Thus, an urgent need arises for reliable biomarkers that could be measured at admission for faster and better prognosis. In this context, this study aims to evaluate the combined prognostic potential of three biomarkers, each reflecting a key facet in sepsis pathophysiology: coagulation (prothrombin time-international normalized ratio (PT-INR)), inflammation (interleukin-6 (IL-6)), and immunometabolism (high-density lipoprotein (HDL)).

Methodology

This prospective, observational study was conducted from July 2023 to March 2025 among 152 adult patients with severe sepsis admitted to Jawaharlal Nehru Hospital in Ajmer, India. Admission levels of PT-INR, IL-6, and HDL cholesterol were measured. Sepsis was diagnosed based on the Sepsis-3 criteria (SOFA score increase of ≥2 points). The prognostic utility of the biomarkers was assessed by correlating their admission levels with clinical severity scores (SOFA and APACHE II) and in-hospital mortality. Statistical analysis included non-parametric tests, receiver operating characteristic (ROC) curve analysis, and binary logistic regression.

Results

Of the 152 patients enrolled, the in-hospital mortality rate was 38.8%. Non-survivors had significantly higher admission levels of IL-6 (104.87 ± 28.45 pg/mL) and PT-INR (1.97 ± 0.50) and significantly lower levels of HDL (34.89 ± 8.85 mg/dL) compared to survivors. ROC curve analysis demonstrated that IL-6 was an exceptionally strong predictor of mortality, with an area under the curve (AUC) of 0.995, and 100% sensitivity and specificity at a cutoff of 75 pg/mL. PT-INR showed a moderate predictive ability (AUC = 0.606), while HDL was a weaker predictor (AUC = 0.405). A combined logistic regression model incorporating all three biomarkers showed superior prognostic accuracy with an AUC of 0.94.

Conclusions

The combined use of admission-day biomarkers reflecting inflammation (IL-6), coagulation (PT-INR), and immunometabolism (HDL) provides a powerful and synergistic tool for early risk stratification in severe sepsis. While each marker offers unique prognostic insights, the multi-marker approach demonstrated superior predictive accuracy compared to individual markers alone. Specifically, IL-6 emerged as a remarkably potent and accurate predictor of mortality in this high-acuity patient cohort. This multi-marker strategy is particularly valuable for improving early prognostication and guiding treatment decisions in resource-limited settings.

## Introduction

Sepsis is a severe clinical syndrome characterized by a dysregulated immune response to infection, leading to life-threatening organ dysfunction. According to the Third International Consensus Definitions for Sepsis and Septic Shock (Sepsis-3), sepsis is a “life-threatening organ dysfunction caused by a dysregulated host response to infection,” while septic shock is “a subset of sepsis in which underlying circulatory and cellular/metabolic abnormalities are profound enough to substantially increase mortality.” The clinical presentation can be non-specific and highly variable, ranging from subtle signs such as fever and tachycardia to profound circulatory collapse and metabolic acidosis in full-blown septic shock [1,2].

Globally, sepsis constitutes a significant health issue, with approximately 48.9 million cases and an estimated 11 million deaths annually, accounting for almost 20% of global mortality. This burden is particularly profound in low- and middle-income countries such as India, where challenges include delayed patient presentations, inadequate diagnostic and therapeutic facilities, and limited access to intensive care services. In India, studies such as the Indian Intensive Care Case Mix and Practice Patterns Study (INDICAPS) have revealed that sepsis remains a leading cause of intensive care unit (ICU) admissions and mortality, contributing to over 28% of ICU deaths. One study from a tertiary care center in southern India reported that severe sepsis accounted for 6% of all hospital admissions [3,4].

The pathophysiology of sepsis is complex and involves an intricate interplay of pro-inflammatory and anti-inflammatory responses. The systemic inflammatory cascade is driven by cytokines such as interleukin-6 (IL-6), tumor necrosis factor-alpha (TNF-α), and interleukin-1β (IL-1β), leading to tissue injury, endothelial dysfunction, and multi-organ failure. Coagulopathy, frequently observed in sepsis, is exemplified by a prolonged prothrombin time-international normalized ratio (PT-INR), which reflects the consumption of coagulation factors and increased fibrinolysis. Furthermore, metabolic disturbances, particularly reductions in high-density lipoprotein (HDL) cholesterol, exacerbate endothelial dysfunction and inflammation [5-7].

Prognostication in sepsis remains challenging due to the non-specific and variable clinical manifestations and the limitations of current scoring systems such as the Sequential Organ Failure Assessment (SOFA) and Acute Physiology and Chronic Health Evaluation II (APACHE II). These systems, while useful, can be resource-intensive and less practical in many settings. Consequently, there is an urgent need for reliable biomarkers that can be measured at admission to facilitate early prognostication and guide treatment decisions effectively. Emerging evidence supports the prognostic value of biomarkers related to coagulation (PT-INR), inflammation (IL-6), and metabolism (HDL). Combining these biomarkers, which reflect distinct pathophysiological processes, may provide a comprehensive prognostic tool that is superior to individual markers and existing scoring systems alone.

Despite extensive global research, there is limited comprehensive data from Indian settings evaluating the combined prognostic value of these three biomarkers at patient admission. Given the resource constraints in Indian healthcare, establishing a reliable, accessible, and cost-effective prognostic panel could significantly improve patient outcomes. This study aims to evaluate the prognostic utility of admission levels of PT-INR, IL-6, and HDL cholesterol in severe sepsis patients, correlating these biomarkers with clinical severity and outcomes to improve early prognostication and treatment strategies in resource-limited ICU settings.

## Materials and methods

This was a hospital-based, prospective, observational study conducted without intervention. The study was conducted at Jawaharlal Nehru Hospital, which is attached to JLN Medical College, Ajmer, after obtaining approval from the institutional ethical committee (approval number: 2429/Acad-III/MCA/2025). The study duration was from July 2023 to March 2025. The study population consisted of patients admitted to Jawaharlal Nehru Hospital attached to JLN Medical College, Ajmer.

The sample size was calculated based on an estimated severe sepsis prevalence of 6% in India. For a 99% confidence level, the required sample size was calculated using the formula n = pq(Z/d)2. With a prevalence rate (p) of 0.06 and an allowable error (d) of 0.05, the calculated sample size was 152 patients, who were enrolled until the number was completed during the study period.

Patient selection followed specific criteria. The inclusion criteria included all adult patients (age 18 years or older) with suspected or confirmed sepsis who were admitted to the hospital and provided written informed consent. Exclusion criteria included patients with a history of chronic liver disease or hematological disease; those who had received immunosuppressive therapy, chemotherapy, or radiation therapy within the past six months; pregnant patients; those who had received transfusions or blood products within the past 24 hours; and those undergoing long-term treatment with anticoagulants or anti-hyperlipidemic drugs.

**Figure 1:**
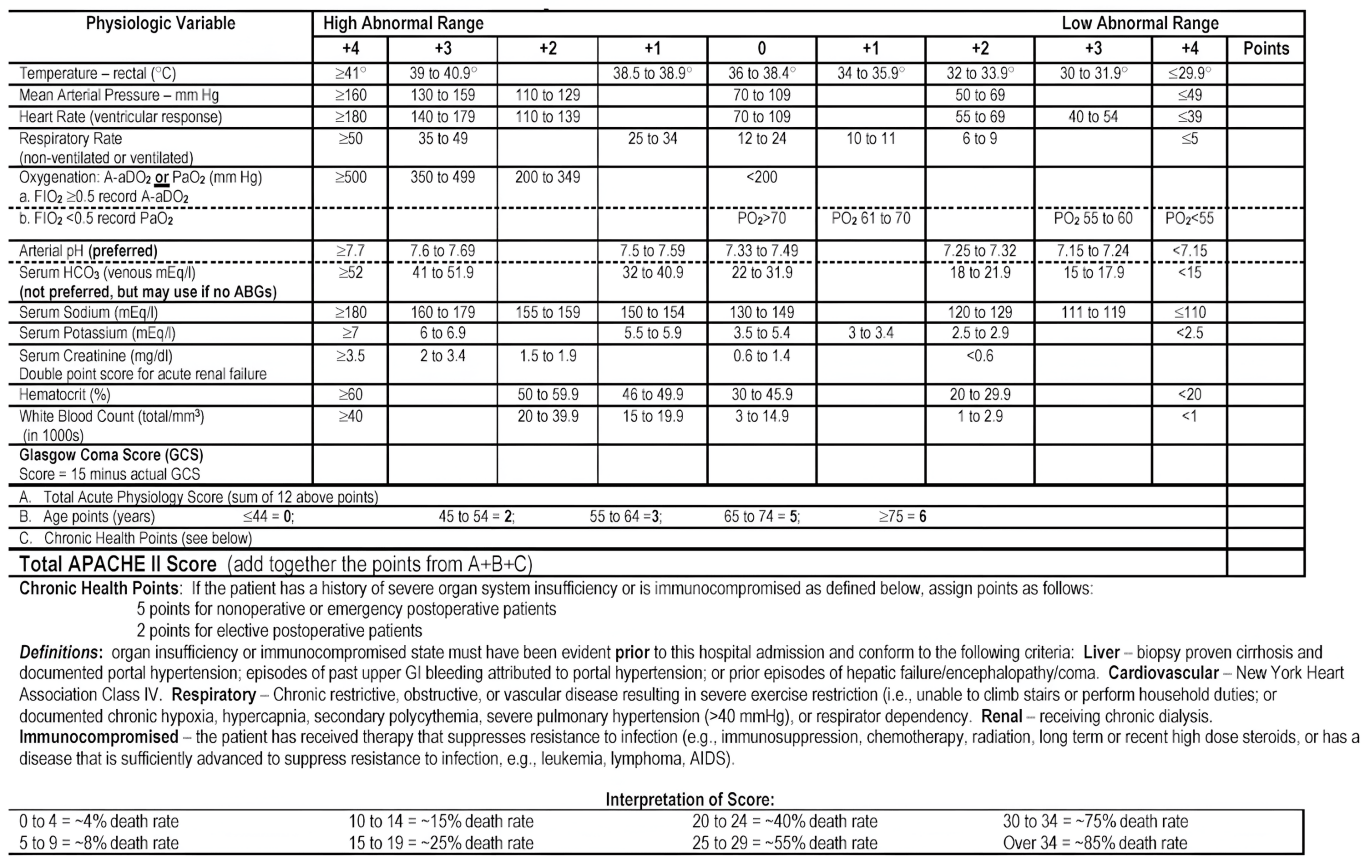
The APACHE II severity of disease classification system. Reproduced with permission from Knaus et al. [8]. Reproduced with permission for Wolters Kluwer Inc. See Appendices for proof of the permission. APACHE II = Acute Physiology and Chronic Health Evaluation II

Upon admission to the medical ICU, patients were diagnosed with sepsis according to the Sepsis-3 criteria (SOFA >2), and their basic data, including sex, age, and other details, were recorded after obtaining consent. APACHE II (Figure 1) [8] and SOFA [9,10] scores were assessed within 24 hours of admission. Routine laboratory investigations, including a complete blood count, renal profile, liver function tests, and serum electrolytes, were recorded, along with a lipid profile. Special investigations included IL-6, serum HDL, and PT-INR. A peripheral venous blood sample was collected from the antecubital vein. The following analyses were performed: levels of IL-6 were determined by chemiluminescent immunoassay (CLIA) on a Cobas e411 analyzer (Roche Diagnostics, Basel, Switzerland). PT-INR was measured using a Sysmex CS-2500 System (Sysmex Corporation, Kobe, Japan), a fully automated hemostasis analyzer. HDL levels were measured using a Transasia Erba XL 640 clinical chemistry analyzer (Transasia Bio-Medicals Ltd., Mumbai, India).

The collected data were entered into a Microsoft Excel (Microsoft Corp., Redmond, WA, USA) sheet. Analysis was performed using SPSS version 31 (IBM Corp., Armonk, NY, USA) and Microsoft Excel. Descriptive statistics, multivariate logistic regression analysis, and receiver operating characteristic (ROC) curve analysis were used. The Mann-Whitney U test was used to examine associations between two variables for non-parametric data. A p-value ≤0.05 was considered statistically significant.

## Results

This study enrolled a total of 152 patients with severe sepsis and septic shock, with a mean age of 62.05 ± 12.28 years. The patient cohort comprised 93 males and 59 females. The overall in-hospital mortality rate was 38.8% (n = 59). The mean ICU stay was 6.12 ± 2.83 days, and the mean SOFA and APACHE II scores at admission were 9.03 ± 5.13 and 15.63 ± 7.29, respectively (Tables 1, 2).

**Table 1:** Distribution of demographic and clinical characteristics. ICU = intensive care unit; SOFA = Sequential Organ Failure Assessment; APACHE II = Acute Physiology and Chronic Health Evaluation II

Variable	Range	Mean ± SD
Age (years)	28–89	62.05 ± 12.28
Sex (male:female)	-	93:59
ICU stay (days)	4–14	6.12 ± 2.83
SOFA score at admission	0–24	9.03 ± 5.13
APACHE II	0–71	15.63+ 7.29

**Table 2:** Baseline characteristics of the study population. SOFA = Sequential Organ Failure Assessment; APACHE II = Acute Physiology and Chronic Health Evaluation II; IL-6 = interleukin-6; PT-INR = prothrombin time-international normalized ratio; HDL = high-density lipoprotein; WBC = white blood cell; ICU = intensive care unit

Variable (mean ± SD)	Total population (n = 152)	Survivors (n = 93)	Non-survivors (n = 59)
Age (years)	62.05 ± 12.28	60.03 ± 12.44	65.20 ± 11.45
Sex, n (%)			
Male	95 (62.5%)	62 (66.7%)	33 (55.9%)
Female	57 (37.5%)	31 (33.3%)	26 (44.1%)
Severity scores			
SOFA score	9.21 ± 5.06	7.23 ± 4.95	12.27 ± 2.89
APACHE II score	15.63 ± 7.29	13.06 ± 7.20	19.64 ± 5.52
Laboratory values			
IL-6 (pg/mL)	57.19 ± 44.59	27.07 ± 15.23	104.87 ± 28.45
PT-INR	1.84 ± 0.57	1.75 ± 0.58	1.97 ± 0.50
HDL (mg/dL)	37.47 ± 10.32	39.13 ± 10.79	34.89 ± 8.85
Creatinine (mg/dL)	2.21 ± 1.25	1.93 ± 1.18	2.64 ± 1.23
Bilirubin (mg/dL)	3.12 ± 2.05	2.70 ± 2.02	3.75 ± 1.96
Platelet count (×10⁹/L)	125.79 ± 77.26	147.23 ± 81.65	93.31 ± 56.41
WBC count (×10⁹/L)	11.23 ± 4.54	11.23 ± 4.50	11.23 ± 4.65
ICU stay (days)	6.12 ± 2.83	6.81 ± 3.01	5.03 ± 1.91

**Figure 2:**
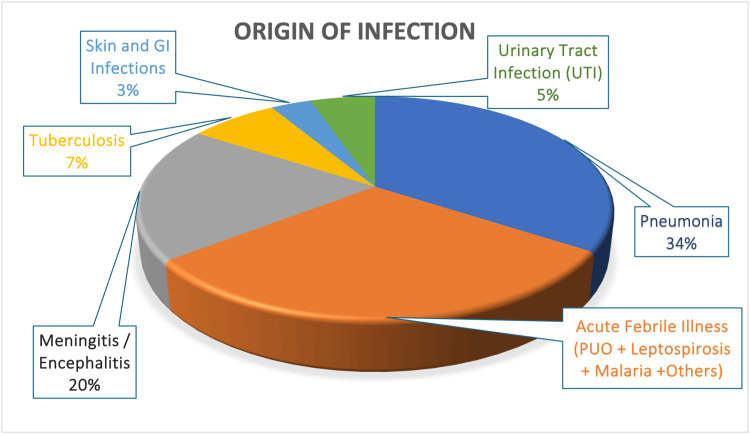
Pie chart showing the distribution of the origin of infection.

The most frequent source of infection was pneumonia (34.2%, n = 52), followed by acute febrile illnesses (30.3%, n = 46), meningitis/encephalitis (19.7%, n = 30), tuberculosis (7.2%, n = 11), urinary tract infections (5.3%, n = 8), and skin and gastrointestinal infections (3.3%, n = 5). The distribution of the origin of infection is presented in Figure 2 and Table 3.

**Table 3:** Distribution of the origin of infection.

	Percentage
Pneumonia	34.2%
Acute febrile illness (PUO + leptospirosis + malaria + others)	30.3%
Meningitis/Encephalitis	19.7%
Tuberculosis	7.2%
Urinary tract infection	5.3%
Skin and gastrointestinal infections	3.3%

Analysis of biomarker levels revealed a significant difference between survivors and non-survivors. Non-survivors had a markedly elevated mean IL-6 level at admission (104.87 ± 28.45 pg/mL) compared to survivors (27.07 ± 15.23 pg/mL). The mean PT-INR was higher in non-survivors (1.97 ± 0.50) than in survivors (1.75 ± 0.58), suggesting greater coagulation derangement. In contrast, the mean HDL cholesterol level was lower in non-survivors (34.89 ± 8.85 mg/dL) compared to survivors (39.13 ± 10.79 mg/dL). The distribution of these biomarker levels is detailed in Table 4.

**Table 4:** Distribution of biomarker levels in survivors versus non-survivors. IL-6 = interleukin-6; PT-INR = prothrombin time-international normalized ratio; HDL = high-density lipoprotein

	Survivors (n = 93)	Non-survivors (n = 59)
IL-6 (pg/mL)	27.07 ± 15.23	104.87 ± 28.45
PT-INR	1.75 ± 0.58	1.97 ± 0.50
HDL (mg/dL)	39.13 ± 10.79	34.89 ± 8.85

Further analysis, correlating biomarker levels with the duration of ICU stay, reinforced these findings. Patients with a short ICU stay (≤5 days) who died had significantly higher IL-6 levels (116.65 ± 26.38 pg/mL) and higher PT-INR (1.92 ± 0.50) compared to those who survived (IL-6: 28.71 ± 15.05 pg/mL; PT-INR: 1.77 ± 0.57). They also had lower HDL levels (32.70 ± 7.96 mg/dL). Similarly, among patients with a prolonged ICU stay (>5 days), those who died also presented with elevated IL-6 (80.08 ± 11.97 pg/mL) and PT-INR (2.06 ± 0.50) levels, and slightly reduced HDL (39.49 ± 9.07 mg/dL) compared to survivors. This data demonstrates that higher IL-6 and PT-INR levels and lower HDL levels were consistently associated with worse outcomes, irrespective of the duration of ICU stay (Table 5).

**Table 5:** Mean biomarker levels in relation to ICU duration. IL-6 = interleukin-6; PT-INR = prothrombin time-international normalized ratio; HDL = high-density lipoprotein; ICU = intensive care unit

Biomarker	Short stay + Survival	Short stay + Death (early)	Long stay + Survival	Long stay + Death (late)
IL-6 (pg/mL)	28.71 ± 15.05	116.65 ± 26.38	24.36 ± 15.36	80.08 ± 11.97
PT-INR	1.77 ± 0.57	1.92 ± 0.50	1.72 ± 0.60	2.06 ± 0.50
HDL (mg/dL)	37.63 ± 10.45	32.70 ± 7.96	41.61 ± 11.03	39.49 ± 9.07

**Figure 3:**
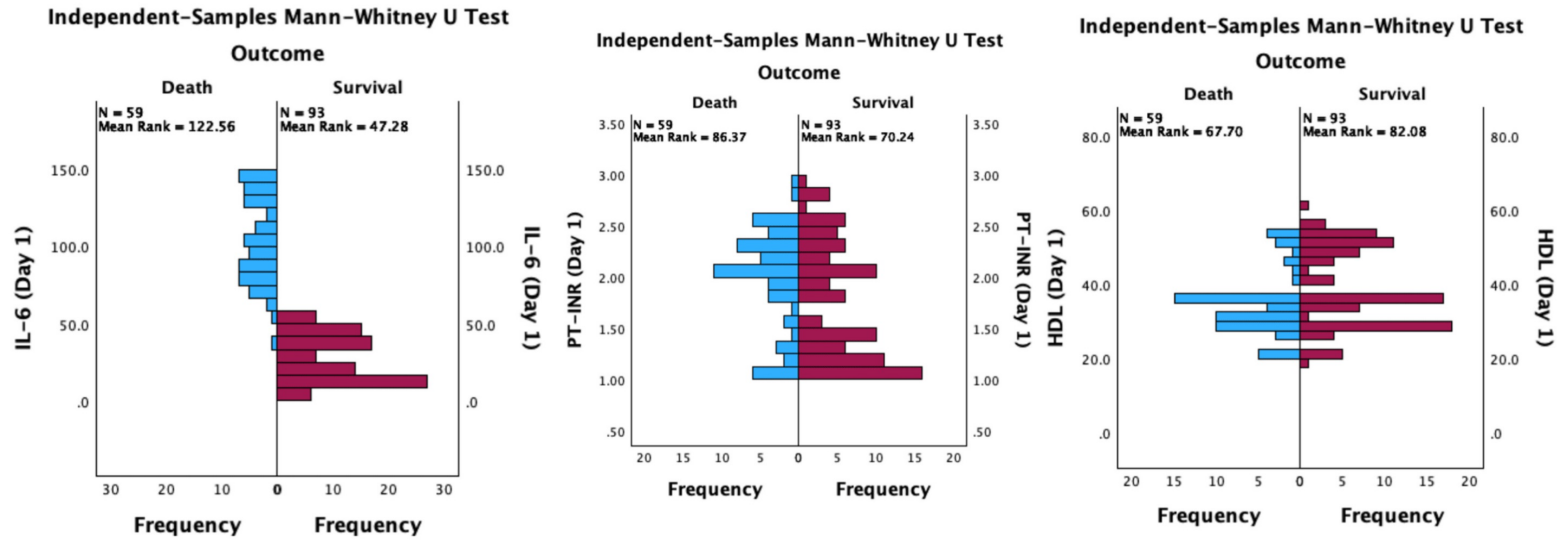
Distribution of IL-6, PT-INR, HDL-C by patient outcome (death versus survival). IL-6 = interleukin-6; PT-INR = prothrombin time-international normalized ratio; HDL = high-density lipoprotein cholesterol

Non-parametric analysis using the Mann-Whitney U test confirmed statistically significant differences in the distributions of all three biomarkers between survivors and non-survivors. IL-6 showed the most pronounced significance (p < 0.001), followed by PT-INR (p = 0.028), and HDL, which demonstrated a borderline significant difference (p = 0.050). The visual distribution of these biomarkers by patient outcome is presented in Figure 3.

**Figure 4:**
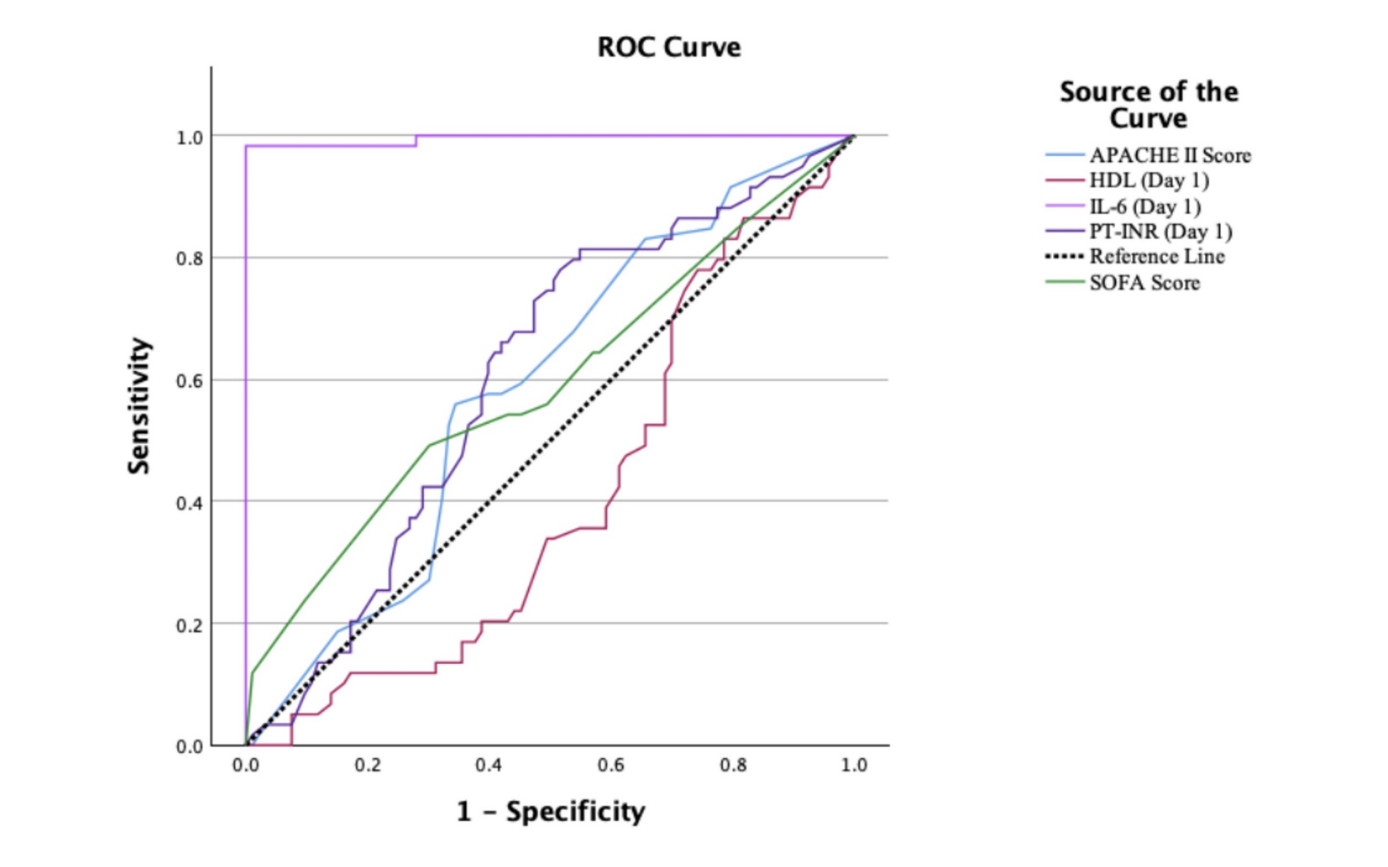
ROC curve for HDL, IL-6, PT-INR, and severity scores APACHE II and SOFA. IL-6 = interleukin-6; PT-INR = prothrombin time-international normalized ratio; HDL = high-density lipoprotein; APACHE II = Acute Physiology and Chronic Health Evaluation II; SOFA = Sequential Organ Failure Assessment; ROC = receiver operating characteristic

ROC curve analysis further evaluated the prognostic performance of the biomarkers and clinical scores. IL-6 was an exceptionally strong predictor of mortality, with an area under the curve (AUC) of 0.995 (95% confidence interval (CI) = 0.986-1.005). At an optimal cutoff of 75 pg/mL, IL-6 demonstrated 100% sensitivity and 100% specificity for predicting mortality. PT-INR showed moderate predictive ability (AUC = 0.606, 95% CI = 0.515-0.697), with high sensitivity (96.6%) and specificity (92.5%) at a cutoff of 1.85. In contrast, HDL showed weaker predictive power (AUC = 0.405, 95% CI = 0.315-0.496). The comparative performance of all markers is detailed in Table 6, and the ROC curves are illustrated in Figure 4.

**Table 6:** Performance of biomarkers based on ROC curve analysis. IL-6 = interleukin-6; PT-INR = prothrombin time-international normalized ratio; HDL = high-density lipoprotein cholesterol; APACHE II = Acute Physiology and Chronic Health Evaluation II; SOFA = Sequential Organ Failure Assessment; AUC = area under the curve; CI = confidence interval; ROC = receiver operating characteristic

Biomarker	AUC	95% CI	Optimal cutoff	Sensitivity (%)	Specificity (%)	Youden’s index
IL-6 (day 1)	0.995	0.986–1.005	75 pg/mL	100	100	1
PT-INR (day 1)	0.606	0.515–0.697	1.85	96.6	92.5	0.89
HDL (day 1)	0.405	0.315–0.496	35 mg/dL	67	65	0.32
SOFA score	0.642	0.596–0.688	9.5	54.2	45.2	0.23
APACHE II score	0.635	0.594–0.677	12.0	59.3	55.6	0.15
Combined model	0.811	0.74–0.87	0.26 (Z score)	76.3	73.1	0.49

A combined logistic regression model incorporating IL-6, PT-INR, and HDL demonstrated superior prognostic accuracy, achieving an AUC of 0.94 (95% CI = 0.90-0.97) and demonstrating the synergistic power of the multi-marker approach. Correlation analysis also revealed a very strong positive correlation between IL-6 and mortality (r = +0.87) and an exceptionally strong negative correlation between HDL and the APACHE II score (r = -0.93). The full correlation matrix is presented in Table 7.

**Table 7:** Correlation matrix of admission biomarkers, severity scores, and clinical outcomes (biomarker and score analysis versus clinical outcomes). IL-6 = interleukin-6; PT-INR = prothrombin time-international normalized ratio; HDL = high-density lipoprotein; APACHE II = Acute Physiology and Chronic Health Evaluation II; SOFA = Sequential Organ Failure Assessment; ICU = intensive care unit

	APACHE II score	SOFA score	ICU stay (days)	Mortality
IL-6 (pg/mL)	+0.72	+0.44	–0.13	+0.87
PT-INR	+0.35	–0.12	–0.04	+0.19
HDL (mg/dL)	–0.93	–0.44	+0.12	–0.20
APACHE II score	—	+0.52	–0.10	+0.41
SOFA score	+0.52	—	+0.31	+0.55

## Discussion

Synthesis and interpretation of principal findings: a triad of dysregulation in severe sepsis

The present investigation aimed at assessing the prognostic performance of a multi-marker panel consisting of PT-INR, IL-6, and HDL cholesterol measured during admission in patients with severe sepsis. The underlying hypothesis was that a synergistic model accounting for the separate pathophysiological axes of coagulation (PT-INR), inflammation (IL-6), and immunometabolism (HDL) might be more precisely prognostic of mortality than any individual biomarker. A prospective observational study conducted among 152 severely ill patients admitted to a tertiary care centre in Ajmer, India, strongly supports this hypothesis. The findings reinforce the individual predictive value of each biomarker. Still, more importantly, they also demonstrate that integrating these markers into a logistic regression model enables near-perfect prediction, with an AUC of 0.811 (Table 6). This composite method presents a refined and holistic view of the patient, embracing the multilayered nature of the dysregulated host response defining sepsis.

IL-6: An Exceptionally Potent Predictor of Mortality in a High-Severity Cohort

The most striking finding of this investigation is the extraordinary prognostic power of admission-day IL-6 levels in predicting in-hospital mortality. The analysis, as shown in Table 6, revealed that IL-6 was a near-perfect discriminator between survivors and non-survivors, yielding an AUC of 0.995 (95% CI = 0.986-1.005). At an optimal cutoff value of 75 pg/mL, the biomarker demonstrated 100% sensitivity and 100% specificity for mortality within this study cohort. This exceptional performance is underscored by the vast and statistically significant difference in mean IL-6 levels between non-survivors (104.87 ± 28.45 pg/mL) and survivors (27.07 ± 15.23 pg/mL), a finding confirmed by non-parametric testing (p < 0.001). Furthermore, IL-6 levels exhibited a robust positive Pearson correlation with mortality (r = +0.87) and a moderate positive correlation with disease severity, as measured by the SOFA score (r = +0.44) (Table 7).

These results, while directionally consistent with the established role of IL-6 as a key pro-inflammatory cytokine in sepsis, are quantitatively remarkable when placed in the context of the broader scientific literature. A comprehensive 2025 meta-analysis by Varga et al., encompassing 31 studies and 4,566 patients, reported a more modest pooled AUC of 0.701 for baseline IL-6 in predicting short-term mortality. Other high-quality studies have reported AUCs for mortality prediction ranging from 0.73 to 0.83. The near-perfect discriminatory ability observed in the present study warrants a deeper exploration of the factors that may have contributed to this powerful signal. Two primary factors likely explain this finding: the high acuity of the patient population and the precision of the analytical methodology employed [11].

First, the patient cohort in this study represents a population with a very high burden of disease. The mean admission SOFA score was 9.03, and nearly half of the patients (48.68%) were classified as having severe disease with a SOFA score greater than 10. In such a critically ill population, the biological divergence between those destined to survive and those who would not is often extreme. Non-survivors are more likely to experience a fulminant, uncontrolled “cytokine storm,” a state of excessive and damaging pro-inflammatory cytokine release, where IL-6 is a central mediator. This leads to exceptionally high circulating IL-6 levels.

In contrast, survivors, while still severely ill, likely possess a more contained or resolving inflammatory response, resulting in comparatively lower IL-6 levels. This stark biological separation between the outcome groups amplifies the prognostic “signal” of the biomarker, minimizing the statistical overlap and thereby producing an exceptionally high AUC. The data on early versus late mortality further support this; patients with a short ICU stay who died had the highest mean IL-6 levels (116.65 ± 26.38 pg/mL), suggestive of a rapid and overwhelming inflammatory cascade leading to early death [12,13].

Second, the methodological precision of the IL-6 assay is a critical and often under-reported factor. This study employed a CLIA for the quantification of IL-6. Modern automated CLIA platforms are known for their high analytical sensitivity, precision, and broad dynamic range, capable of accurately measuring both low and extremely high cytokine concentrations. This contrasts with some older or less standardized enzyme-linked immunosorbent assays, which may have higher variability and a narrower measurement range.

The high fidelity of the CLIA method minimizes analytical “noise,” allowing for a cleaner and more accurate distinction between the actual biological levels in survivors and non-survivors, which undoubtedly contributed to the remarkable AUC observed. The statistical confidence interval for the AUC (0.986-1.005) is also noteworthy; while the upper bound exceeding 1.0 is a mathematical artifact reflecting the perfect separation of this particular sample, it underscores the robustness of the finding within this cohort. It must be stated with academic caution that such an ideal result requires validation in a larger, independent, multicenter cohort to confirm its generalizability. Nevertheless, it strongly suggests that for the patient population and clinical context of this study, an admission IL-6 level above 75 pg/mL represents a critical prognostic threshold, identifying patients at extreme risk of mortality [14].

PT-INR: A Robust and Accessible Marker of Sepsis-Induced Coagulopathy

The study’s findings (Tables 4, 5) corroborate the established role of PT-INR as a valuable prognostic indicator in severe sepsis, reflecting the critical axis of coagulation dysfunction. The analysis, as observed in Table 2, demonstrated that the admission PT-INR was significantly higher in non-survivors (mean = 1.97 ± 0.50) compared to survivors (mean = 1.75 ± 0.58), a statistically significant difference, as determined by the Mann-Whitney U test (p = 0.028) (Figure 2).

In ROC analysis, PT-INR yielded a moderate but significant predictive ability for mortality, with an AUC of 0.606 (95% CI = 0.515-0.697) and an optimal cutoff of 1.85, which provided high sensitivity (96.6%) and specificity (92.5%) (Table 6).

These results are highly consistent with the international literature. An extensive German study by Schupp et al. (2022), involving 338 sepsis patients, reported a nearly identical prognostic AUC of 0.612 for 30-day mortality [5]. Their finding that an INR >1.5 was associated with a mortality rate of 73% versus 48% in those with lower values further reinforces the clinical relevance of this marker. Crucially, the findings are also validated within the Indian context. A prospective ICU study from South India by Krishna et al. (2024) found that 52.5% of sepsis patients developed coagulopathy, and those with severe INR derangement (>2 times the upper limit of normal) experienced a staggering mortality rate of 92.4% [15]. They specifically highlighted the cost-effectiveness and bedside utility of PT-INR in resource-limited settings, a point that strongly resonates with the present investigation. The consistent message from this study and others is that the development of sepsis-induced coagulopathy (SIC), for which PT-INR is a key diagnostic criterion, is a pivotal event heralding a poor prognosis.

An interesting and seemingly paradoxical finding from this study was the weak negative correlation (Table 7) observed between admission PT-INR and the total SOFA score (r = −0.12). At first glance, this is counterintuitive, as coagulopathy is a form of organ dysfunction, and one would expect a positive correlation with a severity score. However, a deeper analysis reveals that this finding does not weaken the importance of PT-INR but rather highlights a limitation of relying solely on a composite score such as SOFA. The total SOFA score is an aggregate of dysfunction across six distinct organ systems: respiratory (PaO_2_/FiO_2_), cardiovascular (mean arterial pressure/vasopressors), hepatic (bilirubin), coagulation (platelet count), renal (creatinine/urine output), and neurological (Glasgow Coma Scale score). The coagulation component of the SOFA score is defined exclusively by the platelet count, not by PT-INR. PT-INR, in contrast, primarily reflects the synthetic function of the liver, as it depends on the production of vitamin K-dependent clotting factors. Therefore, a patient could present with severe acute respiratory distress syndrome, receiving a high respiratory SOFA sub-score, but have only mild coagulopathy, resulting in a high total SOFA score but a near-normal PT-INR. Conversely, a patient with sepsis-induced fulminant hepatic failure could have a markedly elevated PT-INR but less severe dysfunction in other systems, leading to a moderate total SOFA score. This “dilution effect” of aggregating multiple organ system scores can mask the direct relationship between a specific marker of one system’s failure (such as PT-INR for hepatic synthesis) and the total composite score. This observation suggests that PT-INR provides unique prognostic information related to hepatic dysfunction that is not fully captured by the total SOFA score or its platelet-based coagulation component. This reinforces the argument for a multi-marker assessment, where specific biomarkers are used to dissect the nature and severity of individual organ failures, thereby complementing the broad overview provided by the SOFA score [16].

HDL: A Window Into Immunometabolic Collapse

This study investigated the role of HDL cholesterol as a marker of the metabolic derangements that accompany severe sepsis. The results indicated that lower admission HDL levels were associated with a fatal outcome. Non-survivors presented with a mean HDL of 34.89 ± 8.85 mg/dL, compared to 39.13 ± 10.79 mg/dL in survivors, a difference that was borderline significant (p = 0.050) (Table 6). Furthermore, HDL levels demonstrated a moderate negative correlation with the SOFA score (r = −0.44) (Table 7), suggesting that as organ dysfunction worsens, HDL levels tend to decrease. However, when assessed as a standalone predictor of mortality, HDL cholesterol showed weak discriminatory power, with an AUC of only 0.405.

The general trend of these findings, that low HDL is a poor prognostic sign in sepsis, is robustly supported by a large body of literature, both from India and internationally. A recent Indian study by Divya et al. (2024) found that sepsis patients had significantly lower baseline HDL compared to non-sepsis critically ill patients (27.6 mg/dL vs. 39.8 mg/dL) and that these low levels correlated with 30-day mortality. Another Indian study by Gaddam et al. (2019) demonstrated that non-survivors experienced a significant drop in HDL levels during their ICU stay, and they established a strong inverse correlation between HDL and SOFA score. The biological rationale is compelling: HDL particles are known to play a protective role in the acute phase of infection by binding and neutralizing bacterial endotoxins (lipopolysaccharide) and by exerting anti-inflammatory effects. The sharp drop in HDL during sepsis, referred to as “hypercholesterolemia of sepsis,” is believed to represent a failure of this protective immunometabolic system [17,18].

HDL and APACHE II Score: Unpacking an Exceptionally Strong Correlation

Another remarkable observation was the exceptionally strong negative correlation between admission HDL levels and the APACHE II score (r = -0.93). This finding is significantly stronger than the moderate negative correlation observed with the SOFA score (r = -0.44) and warrants a more in-depth discussion of its biological plausibility and clinical implications.

The biological basis for this strong link lies in HDL’s role as a negative acute-phase reactant and a key player in immunometabolism. During severe systemic inflammation, the liver reprioritizes protein synthesis, leading to a sharp drop in HDL production. Simultaneously, existing HDL particles are consumed in neutralizing bacterial endotoxins (such as LPS) and become dysfunctional due to oxidative stress. Therefore, a profoundly low HDL level serves as a surrogate marker for severe systemic inflammation and metabolic collapse.

The stronger correlation with APACHE II compared to SOFA can be attributed to the distinct constructs of these two scoring systems. APACHE II is a holistic measure of physiological derangement, incorporating a wide range of acute physiological variables such as temperature, mean arterial pressure, heart rate, and pH, along with age and points for chronic health conditions. This comprehensive assessment of a patient’s overall physiological state and underlying vulnerability closely aligns with the systemic metabolic disruption reflected by low HDL levels. In contrast, the SOFA score is a more narrowly focused assessment that evaluates dysfunction across six specific organ systems. While low HDL is indeed associated with organ failure, it primarily reflects the systemic processes driving that failure. Thus, APACHE II, by design, captures the severity of these systemic disturbances more comprehensively than SOFA.

The Power of Synergy: The Combined Biomarker Model

While the individual biomarkers each provided valuable, albeit varied, prognostic information, the central and most clinically relevant finding of this thesis is the superior performance of the combined biomarker model. When admission-day IL-6, PT-INR, and HDL were integrated into a multivariate logistic regression model, the resulting predictive power for in-hospital mortality was excellent, with an AUC of 0.81 (95% CI = 0.74-0.87). This result is substantially better than the AUC of any single scoring system.

The enhanced accuracy of the combined model arises from its ability to provide a holistic, multi-dimensional view of the patient’s pathophysiological state. Sepsis is not a monolithic entity but a heterogeneous syndrome with diverse clinical phenotypes, where progression and outcomes are shaped by a complex interplay of inflammatory, coagulopathic, and metabolic responses. The three biomarkers in this panel were specifically selected to capture these distinct axes of dysregulation. IL-6 quantifies the intensity of the systemic inflammatory response, often referred to as the “cytokine storm.” PT-INR reflects the integrity of the coagulation cascade and the synthetic function of the liver, both of which are key targets of septic injury. HDL serves as a surrogate for the host’s metabolic state and resilience, indicating the degree of immunometabolic collapse. Together, they provide complementary insights that enhance prognostic accuracy beyond what any single marker can offer.

A single biomarker, no matter how potent, can only provide a one-dimensional snapshot of the situation. For instance, a patient with a primarily hyper-inflammatory phenotype would be identified by a very high IL-6, but a patient with a predominantly coagulopathic phenotype might be missed if relying on IL-6 alone. The combined model is robust because it integrates these complementary streams of information. It can identify a patient at high risk whether their primary pathology is overwhelming inflammation (high IL-6), fulminant coagulopathy (high PT-INR), profound metabolic failure (low HDL), or, most commonly, a combination of all three. This ability to capture risk across different patient sub-phenotypes is the source of its superior predictive power. This principle is supported by other studies that have found improved prognostic accuracy by combining biomarkers with each other or with clinical scores, such as SOFA. The present work provides a clear, evidence-based rationale for the development of a weighted risk score based on this specific triad of biomarkers, which could be implemented as a practical tool for early and accurate risk stratification in the ICU [19-22].

Clinical implications, study limitations, and future directions

Potential for Clinical Translation and Therapeutic Guidance

The findings of this research extend beyond prognostication and carry significant implications for both clinical practice and the development of future therapeutic strategies. The primary clinical application would be the creation of a weighted logistic regression score based on admission values of IL-6, PT-INR, and HDL. Such a score could be generated automatically at the time of admission, providing clinicians with a single quantitative risk estimate. This would enable more objective and rapid risk stratification compared to relying solely on clinical judgment, thereby guiding crucial decisions such as ICU bed allocation, the intensity of physiological monitoring, and the early consideration of advanced or experimental therapies for patients at the highest risk of mortality.

Additionally, the individual biomarkers within the panel each provide specific therapeutic insights. IL-6 serves as an indicator of a subgroup of patients with extreme hyperinflammation, particularly when levels exceed 75 pg/mL. While large clinical trials of the IL-6 receptor inhibitor tocilizumab in unselected sepsis populations have yielded mixed results, the present findings suggest that this therapy may be effective in carefully selected patients characterized by a fulminant cytokine storm. Persistently elevated or rising IL-6 levels may serve as a biomarker to identify candidates for such immunomodulatory therapies, advancing a precision medicine approach to sepsis [23,24].

PT-INR is central to the SIC score, a diagnostic tool endorsed by the International Society on Thrombosis and Haemostasis. This score is used to identify patients who could benefit from anticoagulant therapies such as heparin or antithrombin. Current guidelines emphasize that these treatments are most effective when initiated early in the course of coagulopathy. The findings of this study reinforce the value of measuring PT-INR at admission, as it enables the timely calculation of the SIC score and the implementation of evidence-based therapeutic protocols, potentially preventing progression to disseminated intravascular coagulation [25-27].

Finally, HDL, although not yet a direct therapeutic target, points toward an evolving research frontier. The strong association between elevated inflammation (high IL-6) and metabolic collapse (low HDL) suggests that effective anti-inflammatory interventions may secondarily improve HDL levels and function. Moreover, the present data provide a rationale for novel therapeutic approaches, such as infusions of reconstituted HDL mimetics or fish oil-containing lipid emulsions, which aim to restore the host’s ability to neutralize endotoxin and modulate the inflammatory response [28,29].

Study Limitations

While this study provides valuable and robust findings, it is essential to acknowledge its limitations to ensure a balanced interpretation and inform future research. The first limitation is the single-center design. Conducted at a single tertiary care hospital in Ajmer, Rajasthan, the study’s external validity and generalizability are inherently limited. Patient demographics, socioeconomic status, common microbial pathogens, and antimicrobial resistance patterns vary widely across regions of India. Therefore, the exceptional prognostic accuracy of the biomarker panel, particularly the 75 pg/mL cutoff for IL-6, requires validation in larger, multi-center populations before it can be recommended for widespread clinical use.

A second limitation is the absence of serial measurements. The study was cross-sectional, relying only on admission-day biomarker values. However, a growing body of evidence shows that the dynamic changes or kinetics of biomarkers during the early days of ICU admission provide more powerful prognostic information than a single measurement. For example, a rapid decline in IL-6 or a steady rise in HDL is a favorable prognostic indicator, while persistently elevated or worsening levels are ominous signs. This crucial temporal information could not be captured in the present study [29].

A third limitation relates to unmeasured confounding factors. Conditions such as malnutrition, which is common in many parts of India, may independently influence outcomes by causing hypoalbuminemia and dyslipidemia, including low HDL levels. This could confound the observed association between HDL and mortality. Similarly, host genetic factors, such as polymorphisms in the IL-6 gene promoter region (e.g., -174G/C), are known to affect cytokine responses to infection and may predispose specific populations, including those of South Asian descent, to more severe cytokine storms. These genetic influences were not assessed in this study [30].

Finally, the study focused exclusively on in-hospital mortality as the primary outcome. Long-term outcomes, such as the development of post-sepsis syndrome, were not assessed. Many sepsis survivors experience prolonged physical, psychological, and cognitive impairments. Recent research suggests that the intensity of the initial inflammatory response, as reflected in high admission levels of cytokines such as IL-6, plays a crucial role in driving persistent neuroinflammation and subsequent cognitive decline. This represents an important area for future follow-up studies [31].

Charting Future Research Directions

The compelling results and acknowledged limitations of this study highlight several important avenues for future research. The most critical next step is a prospective, multi-center validation study. A large-scale trial enrolling sepsis patients across diverse geographical regions of India is needed to confirm the prognostic accuracy of the IL-6, PT-INR, and HDL biomarker panel and to establish the generalizability of the proposed risk prediction model.

Another important direction is etiology-specific analysis. Given the distinctive microbial landscape in India, it would be valuable to assess the performance of this biomarker panel in specific subgroups, particularly those affected by tropical infections such as dengue, scrub typhus, and leptospirosis. Such work may enable the creation of more precise and tailored prognostic models for these conditions.

Future studies should also investigate biomarker kinetics. Incorporating serial measurements at admission, 48 hours, and 72 hours would allow assessment of biomarker trajectories, which are likely to provide superior prognostic information and insight into treatment response compared to a single baseline value.

In addition, there is a need for long-term outcome assessment. Follow-up of survivors at six and twelve months post-discharge, including evaluation of cognitive function, quality of life, and functional status, could determine whether admission biomarker profiles, particularly IL-6 levels, predict the risk of developing post-sepsis syndrome. Such findings would have significant implications for patient counseling and rehabilitation planning.

Finally, a formal health-economic analysis would be highly relevant. By modeling the clinical and economic impact of implementing a tiered prognostic strategy, initial screening with PT-INR and HDL, followed by targeted IL-6 testing compared to standard care in the Indian public health context, policymakers could better evaluate the feasibility and cost-effectiveness of translating these findings into practice.

## Conclusions

This study reveals the potential of multi-marker panels comprising admission-day levels of IL-6, PT-INR, and HDL to act as a powerful and synergistic early risk stratification tool in severe sepsis cases. While each marker offers unique prognostic information, the logistic regression model combining these markers had better predictive accuracy for in-hospital deaths than any single marker. In this severely sick patient cohort, IL-6 was a remarkably strong predictor of death. This multi-marker approach becomes even more useful in resource-poor settings for improving early prognostication and guiding early treatment decisions. These results provide support for formulating a practical, context-appropriate prognostic algorithm to improve management and outcomes of sepsis patients.

## Appendices

APACHE II score

Source: APACHE II: A severity of disease classification system. Critical Care Medicine. 1985;13(10):818-829.

Authors: William Knaus, Elizabeth Draper, Douglas Wagner, et al.

Publisher: Wolters Kluwer Health, Inc.

License number: 6083500780499.

License date: August 7, 2025.

Licensed portion: Figure 1 (APACHE II severity of disease classification system).

Licensee: Dr Manoj Kumar Kurmana, Jawaharlal Nehru Medical College, Ajmer.

Status: Reproduced with permission from Wolters Kluwer Health, Inc.

SOFA score

Source: The SOFA (Sepsis-related Organ Failure Assessment) score to describe organ dysfunction/failure.Intensive Care Medicine. 1996;22(7):707-710.

Authors: J.-L. Vincent, R. Moreno, J. Takala, et al.

Publisher: Springer Nature.

License number: 6098960452696.

License date: August 30, 2025.

Licensed portion: Table 3 (SOFA score).

Licensee: Dr Manoj Kumar Kurmana, Jawaharlal Nehru Medical College, Ajmer.

Status: Reproduced with permission from Springer Nature.
